# Röntgendiagnostik bei Frakturen im Kindes- und Jugendalter – Konsensusbericht des Wissenschaftlichen Arbeitskreises der Sektion Kindertraumatologie der DGU

**DOI:** 10.1007/s00113-021-00994-9

**Published:** 2021-03-22

**Authors:** Klaus Dresing, Francisco Fernández, Peter Strohm, Peter Schmittenbecher, Ralf Kraus, Sektion Kindertraumatologie der Deutschen Gesellschaft für Unfallchirurgie (DGU)

**Affiliations:** 1 Klinik Für Unfallchirurgie, Orthopädie Und Plastische Chirurgie, Universitätsmedizin Göttingen, Robert-Koch-Straße 40, 37075 Göttingen, Deutschland; 2 Kindertraumatologie, Klinikum Stuttgart Olgahospital, Stuttgart, Deutschland; 3 Klinik für Orthopädie und Unfallchirurgie, Klinikum Bamberg, Bamberg, Deutschland; 4 Kinderchirurgische Klinik, Städtisches Klinikum Karlsruhe, Karlsruhe, Deutschland; 5 Klinik für Unfallchirurgie und Orthopädie, Klinikum Bad Hersfeld, Bad Hersfeld, Deutschland; 6 Deutschen Gesellschaft für Unfallchirurgie, Berlin, Deutschland

**Keywords:** Indikation zur Bildgebung, ALARA, Kinder, Jugendliche, Konsens, Diagnostic radiology, Childhood, Adolescence, ALARA, Consensus

## Abstract

Seit Jahrzehnten ist die Projektionsradiographie Standard in der Diagnostik von Frakturen und Verletzungen auch bei Patienten im Kindes- und Jugendalter. Bei jeder Untersuchung mit Röntgenstrahlen sollen aber auch individuell Nutzen und Risiko gegeneinander abgewogen werden. Die Sektion Kindertraumatologie der DGU hat zu verschiedenen Aspekten der Bildgebung zu Diagnostik und Verlaufsbeurteilung, zu Einstellungsmöglichkeiten der intraoperativen Bildgebung, zum Röntgen der Gegenseite, zu Polytrauma und CT, zu postoperativen radiologischen Kontrollen und dem Einsatz der Sonographie ein Konsenspapier erarbeitet.

## Abstract (en)

For decades projection radiography has been the standard in the diagnostics of fractures and injuries, including patients in childhood and adolescent; however, each examination with X‑rays should also be based on an individual assessment of the benefits and risks. The pediatric traumatology section of the DGU has developed a consensus paper on various aspects of imaging for diagnostics and assessment of progression, on setting options for intraoperative imaging, on X‑ray imaging of the opposite side, on polytrauma and computed tomography (CT), on postoperative radiological checks and the use of sonography after an accident.

## Einleitung

Die konventionelle Röntgendiagnostik (Projektionsradiographie) wird bei Kindern, Jugendlichen und Erwachsenen standardmäßig bei klinischem Verdacht auf Frakturen und Luxationen, zur Behandlungsdokumentation und zu Verlaufskontrollen eingesetzt. Bei der Altersgruppe der Kinder und Jugendlichen ist beim Einsatz dieser ionisierenden Strahlung immer zu berücksichtigen, ob der diagnostische Nutzen die Beeinträchtigung durch die Röntgenstrahlen überwiegt. Das Prinzip „as low as reasonably achievable“ (ALARA) hat sich weltweit etabliert [5, 7, 8, 11, 13]

Kinder und Jugendliche sind strahlungsempfindlicher aufgrund der höheren Anzahl sich schnell teilender Zellen [3, 4]. Kinder haben mit ihrer längeren Lebenserwartung potenziell länger das Risiko, sekundär als Strahlenfolge Malignome zu entwickeln [2, 8, 10, 11].

Zum jetzigen Zeitpunkt ist die Projektionsradiographie als „Arbeitspferd“ der Diagnostik in der Kindertraumatologie noch nicht zu ersetzen. Die Methoden zur Sonographie des Bewegungsapparates befinden sich derzeit in einer intensiven Evaluationsphase, finden nach und nach Eingang in den täglichen Routinegebrauch und werden in Zukunft sicherlich breite Anwendung finden [1, 4, 6]. Die Magnetresonanztomographie Kernspintomographie als weitere strahlungsfreie Methode zur Darstellung vor allem der Weichteile soll aufgrund ihres immensen Aufwandes, ihrer eingeschränkten Verfügbarkeit und ihrer (noch) fehlenden Möglichkeit, räumliche Darstellungen zu erzeugen, einzelnen Indikationen vorbehalten werden, bei denen es weniger um die Darstellung einer knöchernen Verletzung als deren weichteiligen Begleitverletzungen geht [12, 7].

Strahlenschutz in der Kindertraumatologie bedeutet zum einen Anwendung aller technischen Möglichkeiten zur Reduktion der Strahlenbelastung bei der Anfertigung von Röntgenbildern, zum anderen aber vor allem auch die zielgerichtete Indikationsstellung zur Röntgenuntersuchung [9, 8]. Bei der Indikationsstellung zur Röntgenuntersuchung sind der Radiologe und auch der speziell weitergebildete Kinderradiologe, der letztendlich vielerorts die „rechtfertigende Indikation“ zur Untersuchung mit ionisierenden Strahlen stellt, jedoch unmittelbar auf die Expertise und die konkrete Fragestellung des behandelnden Kindertraumatologen angewiesen. Angesichts der o. g. Brisanz der Anwendung der Röntgenstrahlung am wachsenden Organismus reicht die Begründung „Verlaufskontrolle“ nach einer Fraktur daher nicht aus. Vielmehr bedarf es bei einer solchen Kontrolluntersuchung einer Konsequenz, die der Behandler aus dem Ergebnis zu ziehen bereit und in der Lage ist. Bei jeder Untersuchung mit Röntgenstrahlen sollen individuell Nutzen und Risiko gegeneinander abgewogen werden [13, 3].

## Methodik

Im Rahmen der Arbeitstagung des Wissenschaftlichen Arbeitskreises der Sektion Kindertraumatologie der Deutschen Gesellschaft für Unfallchirurgie im Januar 2020 wurden unter dieser Prämisse verschiedene Aspekte des Einsatzes von Röntgenstrahlen bei Kindern nach einem Unfallereignis diskutiert und konsentiert.

Von den 23 kindertraumatologisch tätigen TeilnehmerInnen arbeiten 16 in Kliniken der Maximalversorgung, 4 in Schwerpunktkrankenhäusern mit überregionalem Traumazentrum, 1 in einem Schwerpunktkrankenhaus mit regionalem Traumazentrum, 2 in Kliniken der Grund- und Regelversorgung mit lokalem Traumazentrum. 12 TeilnehmerInnen hatte eine unfallchirurgische, 11 eine kinderchirurgische Weiterbildung.

Die angesprochenen Problemfelder erheben dabei keinen Anspruch auf Vollständigkeit, keinen Anspruch darauf, jeden Aspekt der Anwendung von Röntgenstrahlung am verletzten Kind zu beleuchten. Sie sollen für die angesprochenen und ähnliche Situationen eine Orientierungshilfe auf der Basis der umfangreichen kindertraumatologischen Erfahrungen der am Konsens beteiligten Mitglieder des Arbeitskreises bieten.

Die Inhalte und Argumentationen der Diskussion und die daraus abgeleiteten Ergebnisse dieses Konsenses sollen im Folgenden vorgestellt werden.

## Ergebnisse

Im Folgenden werden 14 Themen angesprochen, die den Mitgliedern des Arbeitskreises als regelmäßig wiederkehrende Anlässe zu nichtsachgerechter und -fachgerechter oder gar falscher Anwendung von Röntgenstrahlen in der Kindertraumatologie aufgefallen sind. Die Empfehlungen und Bemerkungen zu diesen Themen wurden in der vorliegenden Form von allen Diskussionsteilnehmern als Konsens akzeptiert.

### Allgemeines

#### Ultraschall in der Frakturdiagnostik

Die Ultraschalldiagnostik zum Frakturnachweis bei Kindern und Jugendlichen soll bei Expertise insbesondere am distalen Radius und am proximalen Humerus eingesetzt werden.

##### Bemerkung.

Die derzeitige Studienlage legt nahe, dass der Ultraschall zur Frakturdiagnostik und bei Verlaufskontrollen bestimmter Frakturen einen erheblichen Teil der röntgenologischen Diagnostik abzulösen in der Lage sein wird.

#### Röntgen der Gegenseite

Grundsätzlich soll im Falle einer vermuteten frischen knöchernen Verletzung auch bei unklaren Befunden die unverletzte Gegenseite zum Vergleich aus strahlenhygienischen Gründen **nicht** geröntgt werden.

##### Bemerkung.

Es darf erwartet werden, dass dem kindertraumatologisch Tätigen die dem Alter und der Reife entsprechende normale Röntgenanatomie bekannt ist. Referenzen für entsprechende Vergleichsaufnahmen finden sich zahlreich in der Fachliteratur und im Internet. Das Röntgen der Gegenseite garantiert darüber hinaus nicht das Erkennen der vermuteten Pathologie.

Besondere Situationen wie die Annahme einer kongenitalen Radiuskopfluxation können wegen des häufigen bilateralen Vorkommens selten eine Indikation zum Röntgen der Gegenseite darstellen. Gleiches gilt z. B. für die Planung einer Korrekturosteotomie zur Definition des individuellen Normalbefundes. Zum Verstehen einer akuten Verletzung darf jedoch das Röntgen der Gegenseite nicht herangezogen werden. Bei dringendem Klärungsbedarf soll auf ein anderes Untersuchungsverfahren ausgewichen werden oder ein entsprechendes Lehrbuch herbeigezogen werden.

#### Anzahl der Röntgenebenen

Zur Diagnosestellung soll bei Frakturverdacht grundsätzlich in 2 idealerweise senkrecht aufeinander stehenden Ebenen geröntgt werden.

##### Bemerkung.

Kann schon aus einer Ebene eine klare Operationsindikation gestellt werden, so wird die 2. Ebene erst intraoperativ, schmerzlos in Narkose dokumentiert. Andererseits rechtfertigt der fehlende Nachweis einer Fraktur in einer Röntgenebene nicht den Verzicht auf eine zweite. Dies birgt die Gefahr des Übersehens einer nur in einer Ebene dislozierten Fraktur.

#### Röntgen bei/nach Metallentfernung

*Vor* einer Metallentfernung muss regelhaft die knöcherne Konsolidierung der Fraktur zeitnah zum Operationstermin radiologisch belegt werden.

*Intraoperativ* soll bei Kindern und Jugendlichen dagegen regelhaft auf das Röntgen verzichtet werden. Die Vollständigkeit der Metallentfernung ist im Operationsbericht zu dokumentieren.

*Postoperativ* soll nach *unproblematischem* Operationsverlauf keine weitere Röntgenuntersuchung zur Dokumentation der Vollständigkeit der Metallentfernung – weder stationär noch ambulant – erfolgen.

##### Bemerkung.

Bei komplizierten Metallentfernungen, z. B. überwachsenen Implantaten, kann das intraoperative Röntgen zur Identifikation des Implantats hilfreich, in Einzelfällen sogar unverzichtbar sein, um das operative Gewebetrauma zu reduzieren.

### Diagnostik bei schwer verletzten Kindern und Jugendlichen

#### CT beim Polytrauma

Bei polytraumatisierten Kindern und Jugendlichen (ISS ≥ 16 Pkt.) soll ein CT-Ganzkörper-Scan (Trauma-Scan) nur individualisiert nach Verletzungsmuster durchgeführt werden.

##### Bemerkung.

Auf Kinder bzw. Jugendliche adaptierte CT-Protokolle sollen implementiert sein.

### Intraoperative Anwendung von Röntgenstrahlen

#### Intraoperative Benutzung der Röntgenaperturblende

Die Einblendung am C‑Bogen zur Strahlenreduktion soll **immer** erfolgen.

##### Bemerkung.

Je nach Region und Fragestellung sind Iris- oder Längsblende zu bevorzugen.

#### Fluoroskopie

Die intraoperative kontinuierliche Röntgendiagnostik (Fluoroskopie) ist zu vermeiden.

##### Bemerkung.

Bei nicht zu vermeidender Anwendung, z. B. Instabilitätsprüfung am Ellenbogen, soll sie gezielt und mit sehr strenger Indikationsstellung eingesetzt werden. Mit einer gepulsten Röntgenanwendung werden meist ähnliche Ergebnisse erzielt.

#### Gepulstes Röntgen

Das gepulste Röntgen am C‑Bogen soll standardmäßig **immer** verwendet werden.

##### Bemerkung.

Geräte, die diese technische Möglichkeit des Strahlenschutzes nicht vorhalten, sollen ausgetauscht werden.

#### Intraoperative Vergrößerung

Zur intraoperativen Vergrößerung soll das sog. Postprocessing verwendet werden.

##### Bemerkung.

Die Positionierung des Bildverstärkers soll mithilfe des Lasers erfolgen, um Übersichtsaufnahmen zur räumlichen Orientierung zu vermeiden. Die C‑Bogen-Stellung soll von Anfang so gewählt werden, dass eine intraoperative Verstellung des Abstands zwischen Patient und Bildverstärker nicht erfolgen muss.

#### Operieren auf dem Bildverstärker

Bei Osteosynthesen bei Kindern und Jugendlichen soll auf das Operieren auf dem steril abgedeckten Bildverstärker („auf dem Topf“) aus strahlenhygienischen Gründen verzichtet werden.

##### Bemerkung.

Bei dieser Methode entsteht meist für Patient und Operateur durch Streustrahlung eine unnötige hohe Strahlenbelastung.

### Spezielle Situationen

#### Bohrdrahtosteosynthese bei suprakondylärer Humerusfraktur

Nach Bohrdrahtosteosynthese einer suprakondylären Humerusfraktur sollen grundsätzlich intraoperativ in 2 Standardebenen (a.-p. und streng seitlich) Fragmentstellung, korrekte Artikulation und Lage des Osteosynthesematerials röntgenlogisch dokumentiert werden. Eine weitere Röntgenuntersuchung erfolgt erst vor der Metallentfernung.

##### Bemerkungen.

Zu diesem Zeitpunkt besteht die optimale Möglichkeit, den Ellenbogen schmerzfrei korrekt einzustellen – es resultiert kein „Verdeckungsbild“. Die Aufnahmen sollen im PACS mit Seitenlokalisation gespeichert werden können. Im Operationsbericht sind die Röntgenbefunde zu dokumentieren.

Ist die Dokumentation im PACS nicht gegeben, wird im näheren Umfeld zur Operation, – spätestens innerhalb 1 Woche – eine Röntgendokumentation in 2 Standardebenen durchgeführt. Eine einzige weitere Röntgenuntersuchung erfolgt vor Metallentfernung nach 4(–6) Wochen.

#### Grünholzfraktur

Nach Grünholzfraktur des distalen, metaphysären Radius eines 6‑ bis 10-jährigen Kindes soll nach 1 Woche eine Röntgenuntersuchung in 2 Standardebenen zum Ausschluss einer sekundären Dislokation erfolgen. Nach 4 Wochen erfolgt eine weitere Röntgenuntersuchung ohne Cast-Verband in 2 Standardebenen zur Dokumentation der Konsolidierung.

##### Bemerkung.

Die angegebenen Röntgenkontrollen sollen nur durchgeführt werden, wenn grundsätzlich die mögliche Notwendigkeit einer aktiven Stellungsverbesserung angenommen wird. Die Angaben gelten nicht für die sehr viel häufigeren, stabilen Wulst- und Stauchungsfrakturen des distalen, metaphysären Radius, die keinerlei radiologischer Kontrollen bedürfen.

#### Claviculafraktur

In der Diagnostik einer Schlüsselbeinfraktur kann beim Kind bis ca. zum 10. Lebensjahr bei Nachweis in einer Ebene auf die Anfertigung einer 2. Ebene verzichtet werden, da sich aus dem dann sich möglicherweise anders darstellenden Dislokationsgrad keine therapeutische Konsequenz ergeben würde. Bei der konservativen Behandlung einer diaphysären Clavicula-Fraktur eines 6‑ bis 10-jährigen Kindes erfolgt darüber hinaus standardmäßig keine weitere Röntgenuntersuchung im Verlauf.

##### Bemerkung.

Ohne therapeutische Konsequenz besteht keine Indikation zur radiologischen Verlaufsdokumentation bei Schlüsselbeinfrakturen.

#### Undislozierte, isolierte Tibiaschaftspiralfraktur

Nach undislozierter, isolierter Tibiaschaftspiralfraktur und konservativer Behandlung eines 6‑ bis 10-jährigen Kindes sollen Röntgenuntersuchung(en) nur nach 1 Woche und nach 4 Wochen erfolgen. Nach einer Woche gilt die rechtfertigende Fragestellung dem möglichen Auftreten einer zu behebenden, sekundären Varisierung, nach 4 Wochen der Konsolidierung und zur Einschätzung der Belastbarkeit.

##### Bemerkung.

Weitere Röntgenaufnahmen werden als nicht notwendig eingeschätzt.

## Diskussion

Die SKT hat sich bei der Zusammenstellung der Empfehlungen vom ALARA-Prinzip leiten lassen. Es besteht kein Dissens, dass im akuten Fall die konventionelle Röntgendiagnostik weiter ihren Stellenwert hat. Werden die ionisierenden Strahlen zu Fraktur- und Verlaufsdiagnostik im Kindes- und Jugendalter eingesetzt, sollen die bestehenden technischen Möglichkeiten zur Strahlenreduktion an den Geräten ausgeschöpft werden. Wenn immer möglich, soll die Indikation zum Röntgen hinterfragt werden, und alternative diagnostische Verfahren wie die Ultraschalldiagnostik zum Frakturnachweis bei Kindern und Jugendlichen, so z. B. am distalen Radius und am proximalen Humerus, sollen eingesetzt werden. Ob in der Zukunft der Ultraschall zur Frakturdiagnostik und bei Verlaufskontrollen bestimmter Frakturen einen erheblichen Teil der röntgenologischen Diagnostik ablösen kann, wird sich zeigen.

Die SKT hofft, dass mit diesem Konsens die Diskussion zum Einsatz von Röntgendiagnostik im Kindes- und Jugendalter stimuliert wird.

## Fazit für die Praxis

Die Indikation zum Einsatz von Röntgenstrahlen bei Kindern und Jugendlichen nach Unfall soll kritisch gesehen werden, Alternativen sollen genutzt werden und der Druck zum Einsatz des Röntgens durch medikolegale Einflüsse soll verringert werden.
